# Case Report: Long-term survival with erlotinib for lung cancer with secondary leptomeningeal carcinomatosis

**DOI:** 10.3389/fonc.2026.1739717

**Published:** 2026-05-14

**Authors:** Diego Neira, Paloma Ruiz, Barbara Nuñez, Gonzalo Navarrete, Rodrigo Vasquez, Luis Villanueva, Olga Barajas

**Affiliations:** 1 Department of Medical Oncology, Hospital Clínico Universidad de Chile, Santiago, Chile; 2 Department of Medical Oncology, Hospital San Juan de Dios, Santiago, Chile; 3 Departments of Medicine and Basic and Clinical Oncology and Center for Cancer Prevention and Control (CECAN), Faculty of Medicine, Universidad de Chile, Santiago, Chile

**Keywords:** EGFR tyrosine kinase inhibitor, erlotinib, liquid biopsy, long-term survival, meningeal carcinomatosis, non-small-cell lung cancer, craniospinal radiotherapy

## Abstract

Erlotinib is an epidermal growth factor receptor (EGFR) tyrosine kinase inhibitor, effective as treatment for advanced non-small cell lung cancer; however, no quality evidence of its benefit in leptomeningeal carcinomatosis has been reported. Previous case series suggest that higher doses are more effective, if higher concentrations reach the central nervous system. Here, we present the case of a 42-year-old Chilean patient with *EGFR*-mutant lung cancer and leptomeningeal metastasis. A multimodal treatment was performed with intrathecal chemotherapy, craniospinal radiotherapy and erlotinib (150 mg/day) for 5 years. At the time of this publication, she remained disease-free, and no *EGFR* mutation was detected via liquid biopsy. Further investigation is needed to determine the optimal anti EGFR regimen, the intrathecal chemotherapy protocol, and their sequencing. It is also of particular interest to identify factors associated with disease progression, and which are predictors of a good response to tyrosine kinase inhibitor among such patients.

## Introduction

Epidermal growth factor receptor (*EGFR*) mutations are present in 10%–26% of non-small cell lung cancers (NSCLCs) (1). Erlotinib is an EGFR tyrosine kinase inhibitor (TKI) with demonstrated efficacy as a first- or later-line treatment in the metastatic setting (2–4) as well as for maintenance therapy (5).

Patients with NSCLC with leptomeningeal metastasis (LM) have a very poor prognosis, the reported median survival without effective treatment is 4 to 6 weeks (6). Its management remains challenging as given the absence of strong evidence to guide treatment. CNS-directed EGFR-targeted therapy is needed.

Here, we present the case of a female patient who visited our clinic in 2010 with a communicating hydrocephalus due to LM secondary to *EGFR*-mutant NSCLC (exon 19 deletion). She received intrathecal chemotherapy (ITC) and craniospinal irradiation (CSI) and was started on erlotinib, which has yielded a complete response to date.

## Case description

**Figure 1:**
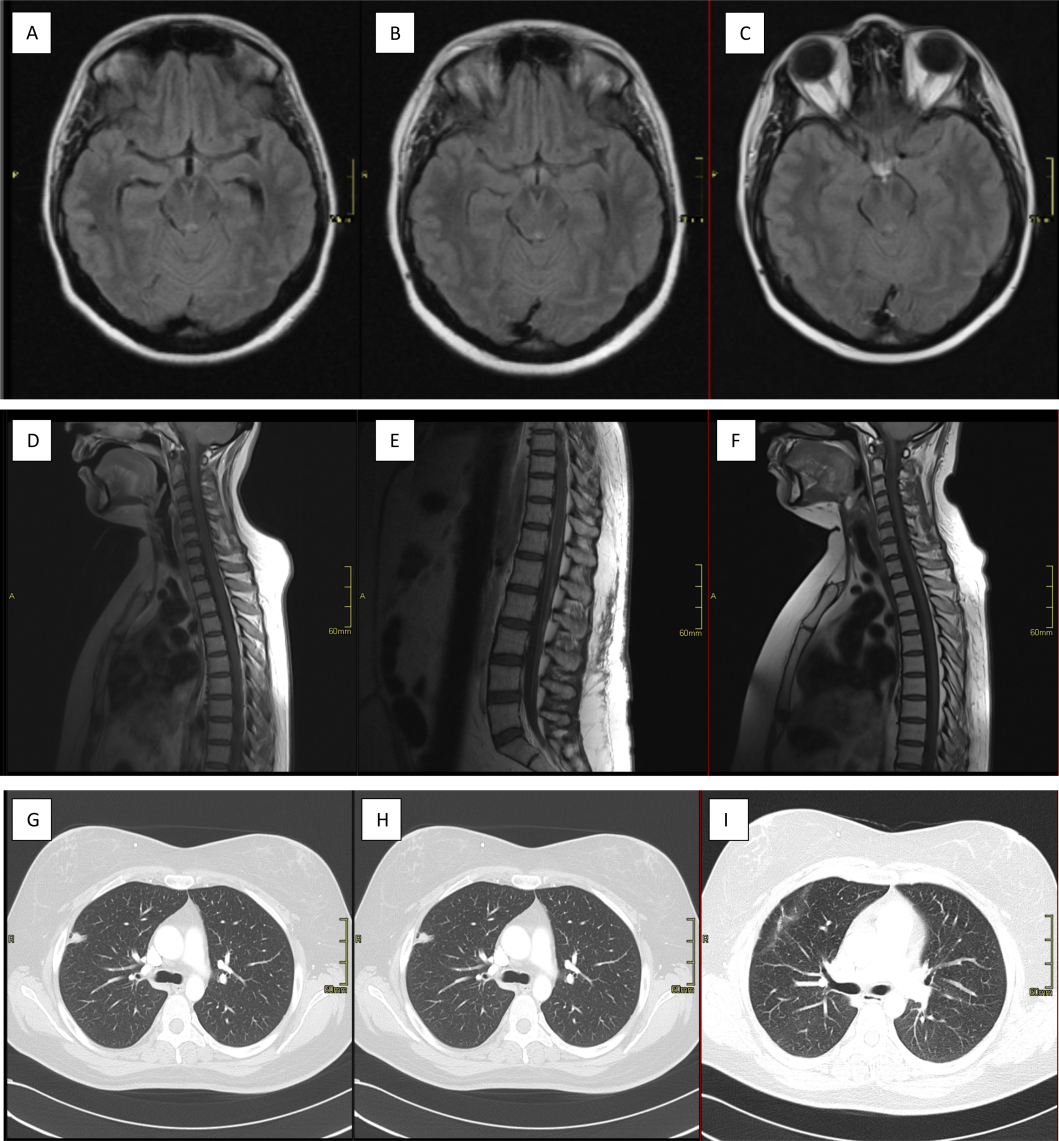
**(A)** decompensated communicating hydrocephalus and an increased signal in the left frontal subcortical white matter at admission. **(B)** Follow-up after intraventricular shunt implantation, showing regression of hydrocephalus and diffuse leptomeningeal involvement. **(C)** Two-and- a half years after diagnosis. Changes in the bifrontal white matter suggestive of leukoencephalopathy is shown. No evidence of current leptomeningeal carcinomatosis or expansion is visible. **(D)** Abnormal leptomeningeal enhancement at the level of the conus and proximal portion of the nerve roots of the lumbar segment at admission. **(E)** Four months later, progression of the leptomeningeal involvement is observed. **(F)** Follow-up after 6 months of craniospinal radiotherapy. The leptomeningeal component has clearly regressed. **(G)** A right upper peripheral lobular opacity of 15 mm in diameter was observed **(H)** Two months later. **(I)** Two-and-a-half years after resection, postoperative cicatricial changes are seen.

A 42-year-old female Chilean patient with long-standing migraine was brought to our emergency department in May 2010 for behavioral disturbances and headache. Due to the presence of bilateral papilledema on the fundus intracranial hypertension was suspected and brain magnetic resonance imaging was performed, which revealed a decompensated communicating hydrocephalus and diffuse leptomeningeal involvement (**Figure 1A**).

As her condition progressed to include areflexic mydriasis and neck stiffness, we inserted a ventriculoperitoneal shunt as well as an external ventricular drain 48 h after she was admitted (**Figure 1B**). We performed a lumbar puncture, which revealed a yielding clear, colorless cerebrospinal fluid, normal glucose levels, leukocytosis and elevated protein. A diagnosis of aseptic meningitis was established and other etiologies including autoimmune, thrombophilia, or infectious disease were ruled out, flow cytometry and presence of neoplastic cells were inconclusive. Computed tomography of the chest, abdomen, and pelvis revealed a non-specific15-mm peripheral focal opacity in the anterior segment of the right upper lobe (**Figure 1G**). Total-spine magnetic resonance imaging showed leptomeningeal enhancement at the level of the conus medullaris and lumbar nerve roots (**Figure 1D**). The patient recovered satisfactorily and was discharge after 18 days.

On chest CT follow-up at in July 2010 revealed that the pulmonary nodule in the right upper lobe had persisted (**Figure 1H**). We decided to resect it using video-assisted thoracoscopy. Analysis of the specimen resulted in a diagnosis of an infiltrating bronchioalveolar carcinoma, with a noninvasive lesion measuring 10 mm in diameter; the surgical margins were positive. A lumbar puncture confirmed the presence of malignant epithelial cells in the cerebrospinal fluid, which we interpreted as leptomeningeal carcinomatosis secondary to lung cancer as clinical symptoms, CSF analysis and radiological findings supported the diagnosis. Therefore, initial TNM classification was pT1aN0M1c, corresponding to stage IVB disease lung cancer.

In August 2010, the tumor board classified the patient as low risk, as no significant neurological deficits were observed, Karnofsky Performance Status (KPS) was greater than 60. Consequently, intra-cerebrospinal fluid (intra-CSF) therapy was initiated using intrathecal methotrexate administered twice weekly, consisting of 12 mg of methotrexate and 4 mg of betamethasone delivered via an Ommaya reservoir catheter. At that time, systemic therapy was not available due to lack of treatment coverage.

After the sixth cycle, we tested the patient’s cerebrospinal fluid, which revealed elevated lactate levels and the presence of neoplastic cells, two additional cycles were administered, for a total of eight cycles completed in early September. A follow-up total-spine MRI imaging demonstrated radiological progression of leptomeningeal carcinomatosis, particularly at the level of the conus medullaris and lumbar nerve roots (**Figure 1E**).

As CSF analysis was continually positive after the eight cycles of ITC and radiological evidence of leptomeningeal progression was observed, we discontinued intra-CSF therapy and initiated palliative CSI followed by systemic therapy (self-funded by the patient), administering 30 Gy in 10 fractions from September to October 2010.

We performed driver mutation analyses on the biopsy sample using the EGFR29 mutation kit, which revealed an *EGFR* exon 19 deletion; therefore, in November 2010 we started the patient on erlotinib (150 mg daily). At the fifth year of follow-up during November 2015, treatment was discontinued by the patient due to the high costs of therapy, as well as the patient’s decision not to continue treatment. Regarding adverse effects during treatment, the patient experienced blepharitis G1, headache G1, neuropathy G1, conjunctivitis G1, polyarthralgia G1, fatigability G1, diarrhea, G1 palmar erythema G1 in the skin folds.

Follow-up was conducted using brain and spinal MRI and chest, abdominal and pelvic CT scans every 3 months for three years and subsequently every 6 months for two years. At 2 years of follow-up, regression of leptomeningeal involvement was evident on MRI and no evidence of distant disease on CT scans (**Figures 1C, F, I**). Currently, surveillance consist of annual clinical examination and chest, abdominal and pelvic imaging.

After the 11th cycle of erlotinib, in conjunction with an acneiform rash G1, the patient’s serum thyroid-stimulating hormone level was 16 mIU/L. We reduced the dosage to 125 mg daily and started levothyroxine 50 mcg daily. The patient remained on erlotinib at same dosage thereafter and disease control was maintained after dose reduction.

At long-term follow up, the patient demonstrated mild, selective memory impairment attributable to late neurotoxicity from craniospinal radiotherapy and intrathecal chemotherapy, while preserving overall functional status and quality of life.

We performed a DNA next-generation sequencing (NGS) Illumina-based assay via liquid biopsy (plasma) in March 2025, which revealed no *EGFR* mutations. A CT of the chest, abdomen and pelvis was performed in October 2025 no evidence of disease.

## Discussion

This case report describes a multimodal treatment with intrathecal chemotherapy, followed by craniospinal radiotherapy and maintenance erlotinib, which yielded survival of 15 years to date, with no evidence of disease.

Phase 3 clinical trials of the treatment of patients with advanced *EGFR*-mutant NSCLC have shown that the use of erlotinib increases progression-free survival and is better tolerated than conventional chemotherapy (2, 3). In the case of patients with LM, evidence that it increases progression-free survival is inconclusive, although a reduction in the size of LM disease has been demonstrated when used at standard doses (7).

In a series of 11 patients with NSCLC and LM who were treated with erlotinib (150 mg daily; n=9) or with gefitinib followed by erlotinib (n=2), Yi et al. (8) administered concomitant therapies such as brain radiotherapy or ITC prior to the TKI dose for patients with *EGFR* mutations or clinical factors predictive of such mutations. The performance status of nine patients improved; however, only six of them (54%) survived for 6 months (8). Morris et al. (9) reported on a retrospective study of 125 patients with NSCLC and secondary LM, concluding that brain radiotherapy was not associated with a difference in overall survival. In contrast, treatment with ITC or TKIs was associated with a longer survival.

This clinical case differs from the series described above because the patient received a multimodal treatment approach including ITC, radiotherapy and erlotinib, which might have influenced her survival as well as a favorable tumor biology.

Notably, the patient presented with a low leptomeningeal disease burden and no evidence of extra–central nervous system involvement. Although no response to chemotherapy was observed in CSF, a clinical complete response was achieved following radiotherapy and subsequently with erlotinib.

Current guidelines recommend that patients with adequate performance status, no major neurological deficits, and limited systemic disease may be managed initially with systemic therapy, radiotherapy, or intra-CSF therapy. In cases of clinical or radiological progression of leptomeningeal disease, or persistence CSF positivity for neoplastic cells, a change in treatment modality is advised.

Importantly, high doses of erlotinib in patients with NSCLC and brain metastases or LM are expected to be more effective than traditional doses, based on the hypothesis that high systemic concentrations translate into high cerebrospinal fluid concentrations (10–13). Researchers have even postulated that poor penetration into the central nervous system when administered at low doses contributes to the acquisition of resistance through mutations such as *T790M* (14).

Variable regimens with erlotinib (weekly, biweekly pulses, etc.) have been described, with survival rates ranging from 12 (12) to 28 months (11). Given that the patient in our case was maintained on low doses of erlotinib, we believe that further studies with other regimens are necessary, keeping in mind that certain pulsatile regimens do not reach the therapeutic range to control lung disease (15).

Osimertinib is a third-generation EGFR inhibitor with significant intracranial activity. In a phase I trial of double-dose osimertinib (160 mg) in patients with LMD from EGFR-mutant lung cancer that had progressed on prior EGFR targeted therapy, 17 of 41 enrolled patients (41 percent) were alive and progression free at 12 months. LM ORR was 62 percent, CSF tumor cell clearence was confirmed in 28 percent and median OS was 11 months (20). Other studies have confirmed activity with osimertinib 80 mg daily, including a study of 22 EGFR T790M-positive patients with LMD in which 52 percent of patients were alive at 18 months (21, 22).

*EGFR* exon 19 deletions are reportedly associated with a better prognosis than exon 21 mutations and wild-type *EGFR* in patients with LM who are treated with TKIs (16), which is consistent with this case. Umemura et al. (16) described the median overall survival in these groups as 11, 7.1, and 1.4 months, respectively, and the median time to symptom progression as 7.8, 2, and 0.9 months, respectively.

*EGFR* mutations may become undetectable in circulating tumor DNA after treatment response, although it is uncommon. One report documented the disappearance of an activating *EGFR* mutation in a patient after treatment with gefitinib and erlotinib (17). Spontaneous regression of tumors with *EGFR* mutations has also been reported (18), although the underlying mechanisms are not entirely clear.

Liquid biopsy is a valuable tool to monitor patients with *EGFR*-mutant NSCLC, allowing assessment of the treatment response and early detection of disease progression and treatment resistance (19). However, further validation in multicenter studies and larger cohorts is required to optimize its clinical use.

This case report has several limitations. It describes a single patient, which may limit generalizability. The initial EGFR assay on lung biopsy did not detect exon 20 insertions, which could have influenced systemic therapy decisions if present. Continuous treatment could not be maintained since 2015 due to financial constraints and patient preference. Post-discontinuation resistance testing was not available, which might have affected management or follow-up strategies. Multimodal treatment carries risk of acute and long-term toxicities, the optimal order of administration as well as its clinical impact remain uncertain according to current evidence. A negative liquid biopsy may reflect low disease burden, assay limitations, or reduced ctDNA shedding.

Finally, access to newer tyrosine kinase inhibitors with improved CNS penetration, such as Osimertinib, was not available at that time; this agent has since been reported to achieve high response rates in similar settings, even associated with chemotherapy. In the leptomeningeal disease subset in FLAURA 2 trial, (n=13) with combination, (n=5) with monotherapy, CNS response rates and complete response were higher with the combination regimen (69 percent and 38 percent) versus Osimertinib (40 percent and 20 percent), respectively (23).

In conclusion, we describe an exceptional case of a patient who has survived in excess of 15 years in response to standard-dose erlotinib for a rare but aggressive lung cancer presentation, with undetectable EGFR mutation after multimodal treatment which is frequently correlating with treatment response. To our knowledge, this is the first report of such a case. Despite the use of third-generation anti EGFR therapies with improved penetration into the CNS, in this setting both response rates and overall survival remain limited. Quality evidence and a consensus on the preferred anti-*EGFR* treatment (or combination with chemotherapy) and recommended dose are absent, as well as either systemic therapy or intrathecal therapy should be initiated upfront.

Further investigation is necessary to determine also which factors are associated with disease progression and which are predictors of a good response to TKIs among patients with NSCLC and LM incorporating for example circulating tumor DNA monitoring during treatment.

Accordingly, dedicated studies are needed in this patient subgroup, given that leptomeningeal disease is an uncommon clinical presentation, associated with an unfavorable prognosis, and has been historically underrepresented in prospective clinical trials.

## Data Availability

The original contributions presented in the study are included in the article/supplementary material. Further inquiries can be directed to the corresponding author.
